# Preoperative Apparent Diffusion Coefficient Values for Differentiation between Low and High Grade Meningiomas: An Updated Systematic Review and Meta-Analysis

**DOI:** 10.3390/diagnostics12030630

**Published:** 2022-03-04

**Authors:** Yueh-Ting Tsai, Kuo-Chuan Hung, Yun-Ju Shih, Sher-Wei Lim, Cheng-Chun Yang, Yu-Ting Kuo, Jeon-Hor Chen, Ching-Chung Ko

**Affiliations:** 1 Department of Medical Imaging, Chi Mei Medical Center, Tainan 710, Taiwan; a80803@mail.chimei.org.tw (Y.-T.T.); 97311151@gms.tcu.edu.tw (Y.-J.S.); a608l1@mail.chimei.org.tw (C.-C.Y.); ytkuorad@gmail.com (Y.-T.K.); 2 Department of Anesthesiology, Chi Mei Medical Center, Tainan 710, Taiwan; ed102605@gmail.com; 3 Department of Hospital and Health Care Administration, College of Recreation and Health Management, Chia Nan University of Pharmacy and Science, Tainan 71710, Taiwan; 4 Department of Neurosurgery, Chi Mei Medical Center, Chiali, Tainan 722, Taiwan; slsw0219@gmail.com; 5 Department of Nursing, Min-Hwei College of Health Care Management, Tainan 736, Taiwan; 6 Department of Medical Imaging, Kaohsiung Medical University Hospital, Kaohsiung 807, Taiwan; 7 Department of Radiological Sciences, University of California, Irvine, CA 92697, USA; jeonhc01@gmail.com; 8 Department of Radiology, E-DA Hospital, I-Shou University, Kaohsiung 824, Taiwan; 9 Department of Health and Nutrition, Chia Nan University of Pharmacy and Science, Tainan 71710, Taiwan; 10 Institute of Biomedical Sciences, National Sun Yat-Sen University, Kaohsiung 804, Taiwan

**Keywords:** ADC, DWI, MRI, meningioma, meta-analysis

## Abstract

The meta-analysis aimed to compare the preoperative apparent diffusion coefficient (ADC) values between low-grade meningiomas (LGMs) and high-grade meningiomas (HGMs). Medline, Cochrane, Scopus, and Embase databases were screened up to January 2022 for studies investigating the ADC values of meningiomas. The study endpoint was the reported ADC values for LGMs and HGMs. Further subgroup analyses between 1.5T and 3T MRI scanners, ADC threshold values, ADC in different histological LGMs, and correlation coefficients (*r*) between ADC and Ki-67 were also performed. The quality of studies was evaluated by the quality assessment of diagnostic accuracy studies (QUADAS-2). A χ^2^-based test of homogeneity was performed using Cochran’s Q statistic and inconsistency index (I^2^). Twenty-five studies with a total of 1552 meningiomas (1102 LGMs and 450 HGMs) were included. The mean ADC values (×10^−3^ mm^2^/s) were 0.92 and 0.79 for LGMs and HGMs, respectively. Compared with LGMs, significantly lower mean ADC values for HGMs were observed with a pooled difference of 0.13 (*p* < 0.00001). The results were consistent in both 1.5T and 3T MRI scanners. For ADC threshold values, pooled sensitivity of 69%, specificity of 82%, and AUC of 0.84 are obtained for differentiation between LGMs and HGMs. The mean ADC (×10^−3^ mm^2^/s) in different histological LGMs ranged from 0.87 to 1.22. Correlation coefficients (*r*) of mean ADC and Ki-67 ranged from −0.29 to −0.61. Preoperative ADC values are a useful tool for differentiating between LGMs and HGMs. Results of this study provide valuable information for planning treatments in meningiomas.

## 1. Introduction

Meningiomas are the most common benign intracranial tumors and account for more than 30% of all brain tumors [1]. The 2016 World Health Organization (WHO) classification of central nervous system tumors divides meningiomas into three grades based on the invasive and histopathological features [2]. Eighty percent of meningiomas are grade I benign meningiomas, referred to as low-grade meningiomas (LGMs); the remaining are grade II atypical meningiomas and grade III malignant meningiomas, referred to as high-grade meningiomas (HGMs) [2]. Most LGMs are slow-growing tumors and usually do not cause clinical symptoms. In contrast, the HGMs show a higher risk of recurrence and lead to higher morbidity and mortality. The recurrence rates were 14.8%, 49.4%, and 69.7% in grades I, II, and III meningiomas, respectively, and overall 5-year survival was 92% for grade I, 78.5% for grade II, and 44% for grade III meningiomas [3,4]. The treatment planning of meningiomas is highly associated with tumor grading. Surgical resection is considered appropriate for LGMs, whereas adjuvant radiotherapy is recommended for HGMs [5,6]. Thus, correct preoperative prediction of tumor grades for meningiomas is crucial in clinical practice.

Diffusion-weighted magnetic resonance imaging (DWI) and apparent diffusion coefficient (ADC) value is a noninvasive, easily accessible technique that has been widely used as a tumor imaging biomarker [7]. It reflects the mobility of water molecules in a specified time, which provides biomedical information of the tumor microstructure [8]. High DWI signal and low ADC value usually indicate the water restriction phenomenon caused by high tumor cellularity [7,8]. Therefore, this technique is widely used to predict tumor characterization noninvasively in several types of brain tumors [9]. Several studies described the correlation of ADC values and the grading of meningiomas [10,11,12,13,14,15]. Some authors reported lower ADC values in HGMs compared to those in LGMs, which highlights that ADC could be a useful imaging marker to differentiate the grades of meningiomas [10,11,12,13,14,15]. However, other studies revealed inconsistent findings [16,17,18,19].

A recent meta-analysis [20] performed a literature search (i.e., Medline and Scopus) in 2019, which demonstrated an overlap in ADC values between LGMs and HGMs despite a higher ADC value in the LGMs, suggesting that ADC may not accurately predict the proliferation potential of the meningiomas. More evidence was presented after 2019 [14,15,19,21,22,23], and updating the latest evidence is imperative. The aim of this meta-analysis was to systematically review the latest evidence from four databases (Medline, Cochrane, Scopus, Embase) and explore the ability of ADC values to differentiate LGMs and HGMs.

## 2. Materials and Methods

### 2.1. Search Strategy

We searched the Medline, Cochrane, Scopus, and Embase databases from their inception dates until January 2022. The Boolean operator “OR” was used to cover similar concepts, while “AND” was used to intersect different concepts. The keywords below were applied to search for eligible records: (“meningioma*” or meningioma [MeSH term]) and (“apparent diffusion coefficient” or ADC or “Diffusion Magnetic Resonance Imaging [MeSH term]” or “diffusion weighted imaging” or DWI). Subject headings (i.e., MeSH terms in Medline) were also adopted in the current literature search. Additional records were screened by reviewing the reference lists of the relevant studies.

### 2.2. Selection Criteria

Prospective or retrospective studies with patients receiving brain MRI evaluation for meningiomas were included. The inclusion criteria were imaging that must have been performed before surgery or treatments, and the results had to include the mean ADC values with standard deviation in low-grade and high-grade meningiomas. Letters, comments, editorials, case reports, proceedings, reviews, and personal communications were excluded.

### 2.3. Study Selection and Data Extraction

The Preferred Reporting Items for Systematic Reviews and Meta-Analyses (PRISMA) 2020 guidelines were applied [24]. The following data were extracted from studies that met the selection criteria: the name of the first author, year of publication, study design, 1.5T or 3T MRI, number of participants in each group, histological tumor grades, mean and standard deviation of ADC values in the LGMs and HGMs, b-values of DWI, ADC measurement methods, threshold values of ADC between LGMs and HGMs, and the correlation coefficient (*r*) between mean ADC and Ki-67 index.

### 2.4. Quality Assessment

The quality of included studies was assessed using the Quality Assessment of Diagnostic Accuracy Studies (QUADAS-2) [25]. The risk of bias in each study was reported as “low,” “unclear” or “high” in the following domains: patient selection, index test, reference standard, and flow and timing. The assessments were performed by two independent reviewers, and a third reviewer was consulted for any uncertainty.

### 2.5. Statistical Analysis

Differences in mean with 95% confidence interval (CI) were calculated for each individual study and for those studies pooled. A χ^2^ test of homogeneity was performed, and the inconsistency index (I^2^) and Q statistics were determined. By assuming heterogeneity across the studies based on a previous meta-analysis [20], a random-effects model was used for outcome assessment, regardless of the finding of statistical heterogeneity. Pooled effects were calculated, and a two-sided *p*-value < 0.05 was considered statistically significant. Sensitivity analysis was evaluated for the outcomes using the one-study-removed test. The analyses were performed using Comprehensive Meta-Analysis (version 3.0) and Review Manager (version 5.4.1) statistical software. For subgroup analysis in ADC threshold values, diagnostic test accuracy and the summary receiver operating characteristics (sROC) were performed using R statistical software (version 4.1.1). Pooled sensitivity, specificity, and area under sROC (AUC) were calculated using the bivariate random-effects model [26,27].

## 3. Results

### 3.1. Literature Search and Study Characteristics

**Figure 1:**
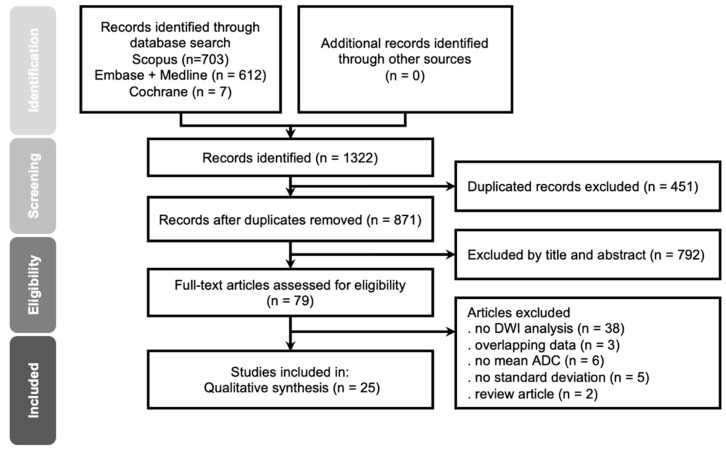
PRISMA flowchart for study selection.

Figure 1 displays the PRISMA flow chart of the study acquisition. Initially, the search of the databases produced 1322 citations. After removing 451 duplicates, 871 publications were screened for eligibility according to the titles and abstracts. The screening process yielded 79 potentially eligible articles for full-text review. A total of 54 studies were excluded due to lack of DWI analysis (n = 38), overlapping data (n = 3), no reported mean ADC (n = 6), no reported standard deviation (n = 5), or review article (n = 2). Finally, 25 studies published from 2001 to 2021, and involving 1552 patients, were included for the present meta-analysis (Figure 1). The baseline characteristics of all included studies are summarized in Table 1. For the included 25 studies, 18 studies were conducted using a retrospective design, while 7 studies were conducted using a prospective design. Sixteen of the 25 studies used 1.5T MRI [10,11,14,15,16,19,21,23,28,29,30,31,32,33,34,35], six studies used 3T MRI [13,22,36,37,38,39], two studies used both 1.5T and 3T MRI [40,41], and one study used 1T MRI [17]. Among the 1552 patients, 1102 patients were diagnosed as LGMs, while 450 patients were diagnosed as HGMs.

### 3.2. Differences in ADC Values between LGMs and HGMs

**Figure 2:**
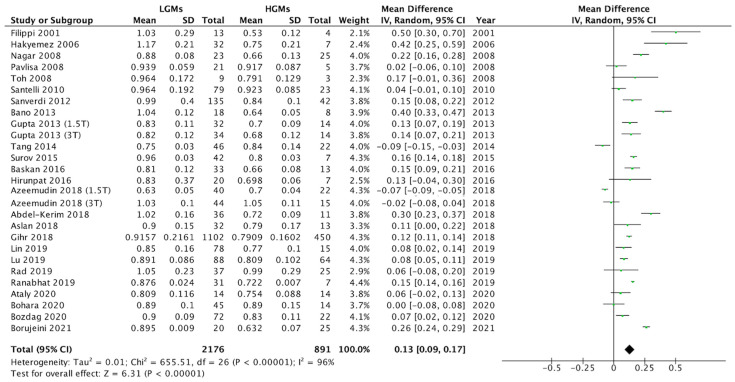
Forest plot for the pooled difference in mean ADC values between LGMs and HGMs. The pooled difference in mean ADC was 0.13 (95% CI = 0.08 to 0.17, *p* < 0.00001).

The difference in ADC means of the two groups are summarized in Figure 2. Heterogeneity of the mean ADC values existed among the 25 studies (Q statistic = 655.51, I^2^ = 96%, *p* < 0.00001). Pooled differences in mean ADC values (0.13, 95% CI = 0.09 to 0.17) indicated that the ADC values were lower in HGMs than in LGMs (*p* < 0.00001, Figure 2), with mean values of 0.79 × 10^−3^ mm^2^/s (95% CI = 0.78 to 0.81) and 0.92 × 10^−3^ mm^2^/s (95% CI = 0.90 to 0.93), respectively.

### 3.3. Subgroup Analysis for 1.5T and 3T MRI Scanners

**Figure 3:**
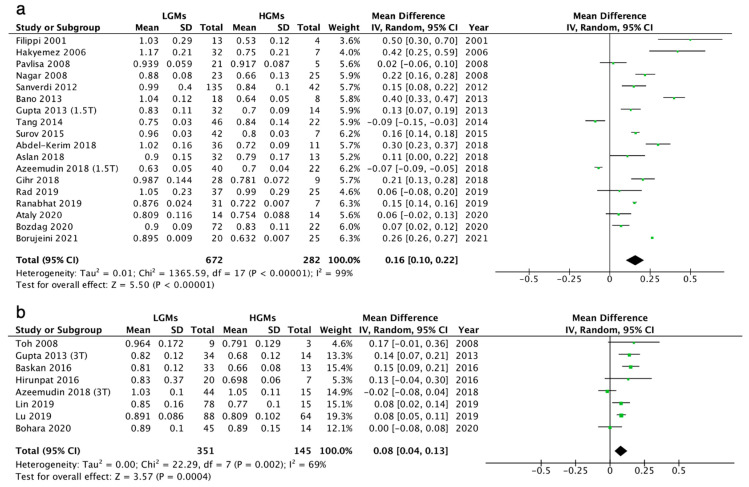
Forest plot for the pooled differences in mean ADC values between LGMs and HGMs in (**a**) 1.5T and (**b**) 3T MRI scanners. The pooled differences were 0.16 and 0.08, respectively (*p* < 0.001).

Both 1.5T and 3T MRI scanners showed significant differences in mean ADC values between LGMs and HGMs (Figure 3). The results of 1.5T MRI scanners showed that LGMs had higher ADC values compared to HGMs (mean difference = 0.16, 95% CI = 0.10 to 0.22, *p* < 0.00001; I^2^ = 99%; 16 studies; n = 954) (Figure 3a). Results for 3T MRI scanners also showed that LGMs also had higher ADC values compared to HGMs (mean difference = 0.08, 95% CI = 0.04 to 0.13, *p* = 0.0004; I^2^ = 69%; 6 studies; n = 496) (Figure 3b).

### 3.4. ADC Threshold Values for Differentiation between LGMs and HGMs

**Figure 4:**
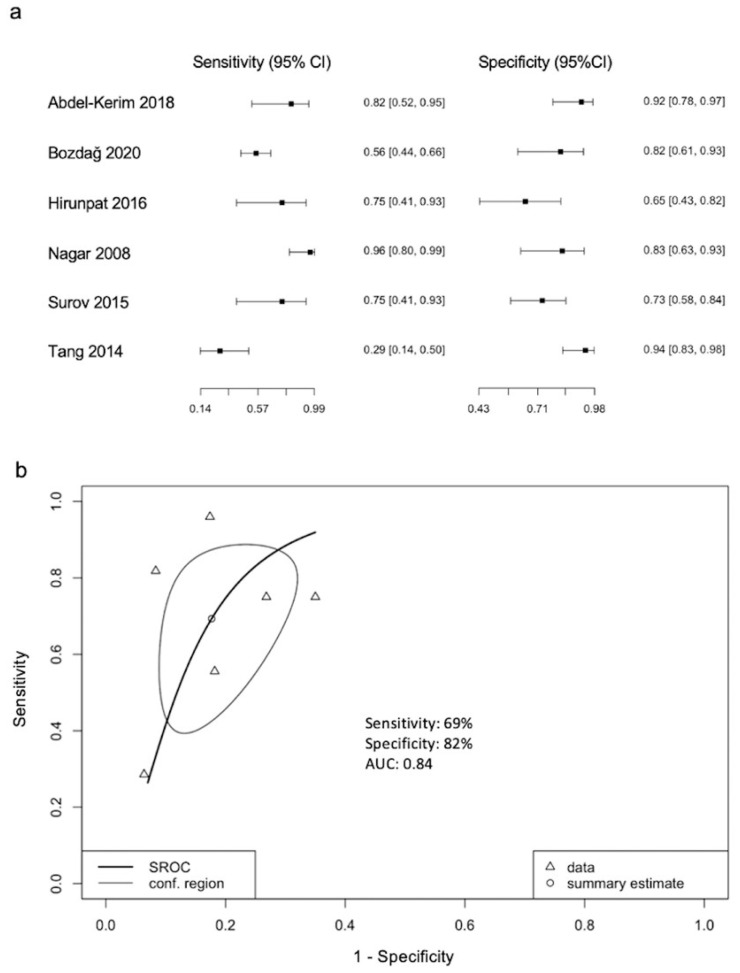
The (**a**) forest plot and (**b**) summary ROC curve in diagnostic test accuracy of ADC threshold values for differentiation between LGMs and HGMs. Pooled sensitivity of 69%, specificity of 82%, and AUC of 0.84 are obtained.

Six of the 25 studies reported threshold ADC values for differentiation between LGMs and HGMs (Table 2) [10,23,29,32,33,37]. The forest plot and sROC curve (Figure 4) of the six studies showed a pooled sensitivity of 69%, specificity of 82%, and AUC of 0.84.

### 3.5. Correlation Coefficients (r) between Mean ADC and Ki-67

Five studies reported a weak-to-moderate inverse correlation between mean ADC values and Ki-67 index in meningiomas [10,13,23,32,34], with the correlation coefficient ranging from −0.28 to −0.61 (Table 3).

### 3.6. Quality Assessment and Sensitivity Analysis

The results of quality assessment for all studies included in this meta-analysis are shown in Supplementary Figure S1, and most studies showed an overall low risk of bias. Sensitivity analysis was performed using one-study-removed analysis in which the meta-analysis of the mean ADC values was performed with each study removed in turn (Supplementary Figure S2). Differences between mean ADC values did not vary markedly with the removal of each study, indicating that the data were not overly influenced by each study.

## 4. Discussion

MRI is widely used for the evaluation of meningioma; however, no broad consensus of conventional MRI findings that can distinguish HGMs from LGMs [42]. The aim of this study was to evaluate the effectiveness of preoperative ADC values for differentiation between low-grade and high-grade meningiomas. The present meta-analysis showed that preoperative mean ADC values were significantly lower in HGMs than in LGMs, and the results were consistent for both 1.5T and 3T MRI scanners. In subgroup analyses, a good AUC was obtained in ADC threshold values for differentiation between LGMs and HGMs. The mean ADC values in different histological LGMs and correlation coefficients (*r*) between mean ADC and Ki-67 were also presented in this study.

According to the WHO 2016 classification, the grading of meningiomas is based on the mitotic number and the invasive features. WHO grade I meningiomas are characterized by less than four mitotic cells per 10 high power fields (HPF), no brain invasion, and less than three of the following atypical features: increased cellularity, necrosis, prominent nucleoli, sheeting, and the high nuclear-to-cytoplasmic ratio [2]. WHO grade I meningiomas encompass nine histological subtypes, including meningothelial, fibroblastic, transitional, psammomatous, angiomatous, microcystic, secretory, lymphoplasmacyte-rich, and metaplastic meningiomas [43]. In contrast, grade II and III meningiomas are defined as having more mitotic cells (≥4) per 10 HPF, or brain invasion, or with more than three atypical features [2]. Three histological subtypes (choroid, clear-cell, and atypical) were identified in WHO grade II meningiomas, and another three subtypes (papillary, rhabdoid, and anaplastic) were found in WHO grade III meningiomas [43].

The Ki-67 index is an important cellular proliferation marker, and a positive correlation between the Ki-67 index and grading of meningiomas was reported [44]. Moreover, a higher Ki-67 index is associated with poor prognosis and a higher risk of tumor recurrence in meningiomas [45]. ADC values were shown to reflect the microstructural cellularity in many tumors [7]. In fact, some authors reported that ADC values correlated inversely with the Ki-67 proliferation index and are helpful in differentiating low and high-grade meningiomas [10,12,13]. Tang et al. [10] reported an ADC value of 0.84 × 10^−3^ mm^2^/s and Ki-67 of 2% in LGMs compared with an ADC of 0.75 × 10^−3^ mm^2^/s and Ki-67 of 9% in HGMs. Five articles in the present meta-analysis reported an association between low ADC value and high Ki-67 in meningiomas [10,13,23,32,34]. Surov et al. [12] reported a threshold ADC value of 0.85 × 10^−3^ mm^2^/s for differentiating between LGMs and HGMs, with positive predictive value (PPV) and negative predictive value (NPV) of 33% and 97%, respectively. Bozdag et al. [23] reported a higher cut-off value of 0.89 × 10^−3^ mm^2^/s with PPV and NPV of 91% and 36% in a larger sample size. We first used six different studies to evaluate the diagnostic test accuracy of threshold ADC values for differentiation between LGMs and HGMs, and we obtained excellent accuracy with an AUC of 0.84. Although most studies reported lower ADC values in HGMs, few studies revealed inconsistent findings [10,41]. Tang et al. [10] and Azeemudin et al. [41] reported higher ADC values in HGMs, which may be explained by the significantly higher ADC values in chordoid meningiomas (WHO grade II) among all LGMs and HGMs [46]. The increased ADC values in the chordoid subtype may be caused by the unique histopathologic features of mucoid stroma and vacuolated cytoplasm, leading to a decreased nucleus-to-cytoplasm ratio [46]. Another possible explanation is that the ADC values are influenced both by perfusion and diffusion effects [47]. HGMs usually have a higher degree of blood volume (perfusion), which results in a higher ADC [10,48].

The present study showed consistent results in both 1.5T and 3T MRI scanners, which means the differences in ADC values between LGMs and HGMs are not affected by different magnetic field strengths of MRI. In six articles investigating the ADC values of different histological LGMs, fibroblastic meningiomas showed the lowest mean ADC value, and angiomatous meningiomas showed the highest mean ADC value. For HGMs, no studies in the available literature reported the ADC values in different histological subtypes. Recently, Meyer et al. [20] reported a meta-analysis reviewing studies from two databases (Medline and Scopus) until November 2019, and the results showed that ADC might not accurately predict proliferation potential and cellularity in the meningiomas, and no validated ADC threshold can be recommended for distinguishing LGMs from HGMs. As compared with 1055 patients reported by Meyer et al. [20], the present study collected the latest studies until January 2022 from four databases (Medline, Cochrane, Scopus, Embase), with a total of 1552 meningioma cases. Six extra updated studies (2019–2022) were included in the present meta-analysis. Meyer et al. [20] reported only weak inverse correlations (*r* = −0.36) existing between ADC and Ki-67 in meningiomas, and significant overlap of ADC values between LGMs and HGMs were observed. In contrast, the present meta-analysis first reported significant differences in ADC values between LGMs and HGMs. In clinical practice, the valuable ADC threshold value for differentiation between LGMs and HGMs was first analyzed in the present study. The discrepancy between the two meta-analyses can be explained by the additional 497 (497/1055, 47.1%) patients collected in the six additional updated studies [14,15,19,21,23,39] in the present meta-analysis and by searching two more databases (Cochrane and Embase). On the other hand, Ugga et al. [49] recently reported a meta-analysis of radiomics and machine learning for the prediction of intracranial meningioma grading based on preoperative MRI, and promising results with an overall pooled AUC of 0.88 was obtained.

Histological grading is the current gold standard in terms of diagnosing and treating meningiomas [50]. Surgery is the classical first-line treatment for all meningiomas. However, a wait-and-see strategy monitored by clinical and MRI follow-up should be considered since most meningiomas (80%) are benign tumors with stable disease status. For LGMs, the aim of surgery may be the relief of the clinical symptoms caused by mass effects. Postoperative adjuvant radiotherapy should be used more conservatively because all radiotherapeutic procedures have long-term side effects and might affect future treatments [51]. However, WHO grade II and III meningiomas are aggressive tumors with high recurrence rates, and aggressive tumor resection during primary surgery combined with adjuvant radiotherapy of the tumor area may be more beneficial for these patients [3,52]. The present study is the first to report significant differences in ADC values between LGMs and HGMs in a meta-analysis with large case numbers. The results provided valuable information for the preoperative diagnosis and planning of treatments in meningiomas.

There are still several limitations in our study. First, publication bias may exist because studies with negative results were less likely to be published and were therefore not included in the meta-analysis. The automatically calculated ADC values may be affected by different vendors, applied b-values, and regions of interest (ROIs) (i.e., single ROI or whole tumor measurement). The overall diagnostic test accuracy of ADC threshold values for differentiation between LGMs and HGMs cannot be obtained due to the lack of comprehensive raw data in each study. Finally, the association between ADC and cellularity in meningiomas was not determined because the updated data reflecting new concepts were not sufficient.

## 5. Conclusions

The present meta-analysis revealed that mean ADC values are significantly lower in HGMs than in LGMs in both 1.5T and 3T MRI scanners. The threshold ADC values also showed excellent accuracy for differentiation between LGMs and HGMs in subgroup analysis. Therefore, preoperative ADC appears to be a useful noninvasive tool for differentiation between LGMs and HGMs. The results of this study have the potential to offer valuable information for the planning of treatments in meningiomas, including the extent of tumor resection, implementation of adjuvant radiotherapy, and the appropriate time intervals for MRI follow-up.

## Figures and Tables

**Table 1:** Baseline characteristics of the 25 studies included in the meta-analysis.

Study	Study Design	MRI	ROI	b Value	LGMs			HGMs		
		Tesla		(s/mm^2^)	Numbers	Mean ADC	SD	Numbers	Mean ADC	SD
					(×10^−3^ mm^2^/s)			(×10^−3^ mm^2^/s)		
Filippi (2001)	Prospective	1.5T	Single	0,1000	13	1.03	0.29	4	0.53	0.12
Hakyemez (2006)	Prospective	1.5T	Single	0,1000	32	1.17	0.21	7	0.75	0.21
Nagar (2008)	Retrospective	1.5T	Single	0,1000	23	0.88	0.08	25	0.66	0.13
Pavlisa (2008)	Prospective	1.5T	Single	0,500,1000	21	0.94	0.06	5	0.92	0.09
Toh (2008)	Prospective	3T	Single	0,1000	9	0.96	0.17	3	0.79	0.13
Santelli (2010)	Retrospective	1T	Single	0,800	79	0.96	0.19	23	0.92	0.09
Sanverdi (2012)	Retrospective	1.5T	Single	0,500,1000	135	0.99	0.4	42	0.84	0.10
Bano (2013)	Prospective	1.5T	Single	0,1000,2000	18	1.04	0.12	8	0.64	0.05
Gupta (2013)	Retrospective	1.5T	Single	0,1000	32	0.83	0.11	14	0.70	0.09
		3T	Single	0,1000	34	0.82	0.12	14	0.68	0.12
Tang (2014)	Retrospective	1.5T	Single	0,1000	46	0.75	0.03	22	0.84	0.14
Surov (2015)	Retrospective	1.5T	Whole	0,1000	42	0.96	0.03	7	0.80	0.03
Baskan (2016)	Retrospective	3T	Single	0,1000	33	0.81	0.12	13	0.66	0.08
Hirunpat (2016)	Retrospective	3T	Single	0,1000	20	0.83	0.37	7	0.70	0.06
Abdel-Kerim (2018)	Prospective	1.5T	Single	0,1000	36	1.02	0.16	11	0.72	0.09
Aslan (2018)	Retrospective	1.5T	Single	0,1000	32	0.90	0.15	13	0.79	0.17
Azeemudin (2018)	Retrospective	1.5T	Single	0,1000	40	0.63	0.05	22	0.70	0.04
		3T	Single	0,1000	44	1.03	0.10	15	1.05	0.11
Gihr (2018)	Retrospective	1.5T	Whole	0,1000	28	0.99	0.14	9	0.78	0.07
Lin (2019)	Prospective	3T	Single	0,1000	78	0.85	0.16	15	0.77	0.10
Lu (2019)	Retrospective	3T	Single	0,1000	88	0.89	0.09	64	0.81	0.10
Rad (2019)	Retrospective	1.5T	Whole	0,1000	37	1.05	0.23	25	0.99	0.29
Ranabhat (2019)	Retrospective	1.5T	Single	0,90,1000	31	0.88	0.02	7	0.72	0.01
Ataly (2020)	Retrospective	1.5T	Single	0,1000	14	0.81	0.12	14	0.75	0.09
Bohara (2020)	Retrospective	3T	Whole	0,1000	45	0.89	0.10	14	0.89	0.15
Bozdag (2020)	Retrospective	1.5T	Single	0,1000	72	0.90	0.09	22	0.83	0.11
Borujeini (2021)	Retrospective	1.5T	Whole	0,500,1000	20	0.90	0.01	25	0.63	0.01
										159

**Table 2:** ADC threshold values for differentiation between LGMs and HGMs.

Study	ADC Threshold Values (×10^−3^ mm^2^/s)	Sensitivity (%)	Specificity (%)	PPV (%)	NPV (%)
Nagar (2008)	0.80	96	82	86	95
Tang (2014)	0.70	29	94	67	75
Surov (2015)	0.85	73	73	33	97
Hirunpat (2016)	0.80	75	65	46	87
Abdel-Kerim (2018)	0.79	81	92	75	94
Bozdag (2020)	0.89	56	82	91	36

PPV: positive predictive value. NPV: negative predictive value.

**Table 3:** Correlation coefficients (*r*) between mean ADC and Ki-67.

Study	*r*
Tang (2014)	−0.34
Surov (2015)	−0.61
Baskan (2016)	−0.33
Gihr (2018)	−0.32
Bozdag (2020)	−0.29

## Data Availability

The original contributions presented in the study are included in the article and Supplementary Materials. Further inquiries can be directed to the corresponding author.

## Supplementary Materials

The following are available online at https://www.mdpi.com/article/10.3390/diagnostics12030630/s1: Supplementary Figure S1: Quality assessment of all included studies. Supplementary Figure S2: Sensitivity study.
