# Supplementary material for: Preoperative Apparent Diffusion Coefficient Values for Differentiation between Low and High Grade Meningiomas: An Updated Systematic Review and Meta-Analysis
Source: Diagnostics (Basel). 2022 Mar 4;12(3):630. doi: 10.3390/diagnostics12030630 (PMC8947055; doi:10.3390/diagnostics12030630)
Supplement: Supplementary file 1 [file diagnostics-12-00630-s001.zip › diagnostics-1609972-supplementary.pdf]

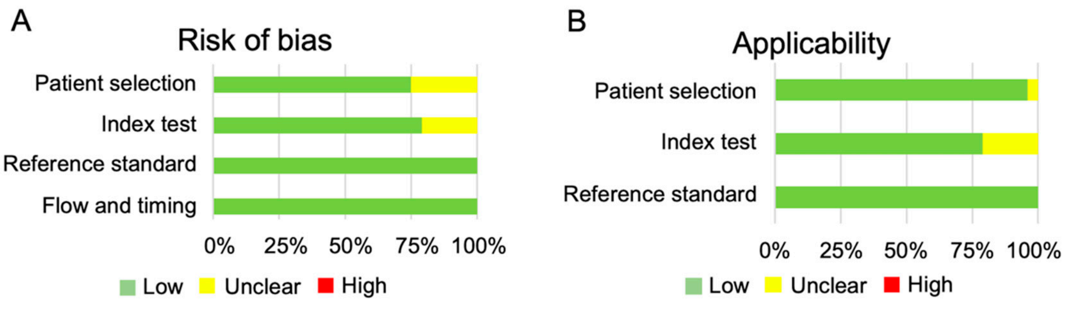

Supplementary Figure S1. Quality assessment of all included studies. Most studies showed an overall low risk of bias

Study name	Difference in means	Standard error	Variance	Lower limit	Upper limit	Z-Value	p-Value
Filippi, 2001	0.105	0.048	0.002	0.011	0.199	2.198	0.028
Hakyemez, 2006	0.105	0.048	0.002	0.012	0.199	2.200	0.028
Nagar V. A, 2008	0.112	0.048	0.002	0.019	0.206	2.352	0.019
Pavlisa G, 2008	0.120	0.049	0.002	0.025	0.215	2.468	0.014
Toh, 2008	0.115	0.048	0.002	0.020	0.209	2.380	0.017
Santelli L, 2010	0.119	0.048	0.002	0.025	0.214	2.468	0.014
Sarıverdi S.E, 2012	0.115	0.048	0.002	0.021	0.210	2.389	0.017
Gupta A, 2013	0.116	0.048	0.002	0.021	0.210	2.405	0.016
Gupta A, 2013	0.115	0.048	0.002	0.021	0.210	2.398	0.016
Bano S, 2013	0.105	0.047	0.002	0.012	0.198	2.218	0.027
Tang Y, 2014	0.125	0.050	0.002	0.028	0.222	2.524	0.012
Surov A, 2015	0.114	0.046	0.002	0.024	0.204	2.494	0.013
Baskan O, 2016	0.115	0.048	0.002	0.021	0.209	2.391	0.017
Hirunpat S, 2016	0.116	0.048	0.002	0.022	0.210	2.412	0.016
Abdel-Kerim, 2018	0.109	0.048	0.002	0.015	0.203	2.281	0.023
Azeemudin A, 2018	0.125	0.052	0.003	0.022	0.227	2.390	0.017
Azeemudin A, 2018	0.122	0.049	0.002	0.027	0.218	2.504	0.012
Gihr GA, 2018	0.113	0.048	0.002	0.019	0.207	2.349	0.019
Aslan K, 2018	0.117	0.048	0.002	0.022	0.211	2.418	0.016
Lu Y, 2019	0.118	0.049	0.002	0.023	0.213	2.426	0.015
Rad H S, 2019	0.119	0.048	0.002	0.024	0.213	2.456	0.014
Ranabhat K, 2019	0.115	0.048	0.002	0.021	0.209	2.404	0.016
Lin L, 2019	0.118	0.048	0.002	0.023	0.213	2.438	0.015
Ataly B, 2020	0.119	0.048	0.002	0.024	0.214	2.453	0.014
Bozdag M, 2020	0.118	0.049	0.002	0.023	0.214	2.439	0.015
Bohara M, 2020	0.121	0.049	0.002	0.026	0.216	2.494	0.013
Borujeini, 2021	0.122	0.025	0.001	0.073	0.170	4.910	0.000
	0.116	0.047	0.002	0.024	0.209	2.459	0.014

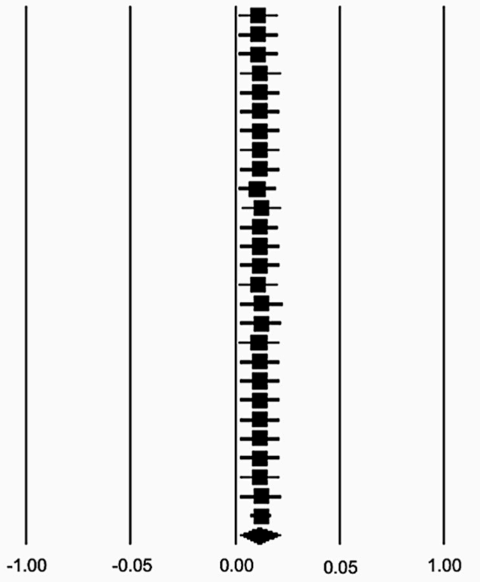

Supplementary Figure S2. Sensitivity study. The differences in mean ADC values did not vary markedly with removal of each study, indicating that the data were not overly influenced by each study.
